# Null effect of ginsenoside Rb1 on improving glycemic status in men during a resistance training recovery

**DOI:** 10.1186/s12970-015-0095-6

**Published:** 2015-08-20

**Authors:** Wei-Hsiang Chang, Ying-Lan Tsai, Chih-Yang Huang, City C. Hsieh, Rungchai Chaunchaiyakul, Yu Fang, Shin-Da Lee, Chia-Hua Kuo

**Affiliations:** Department of Sports Sciences, Laboratory of Exercise Biochemistry, University of Taipei, Taipei, Taiwan; Department of Physical Education, National Hsinchu University of Education, Hsinchu, Taiwan; Department of Athletic Training and Health, National Taiwan Sport University, Taoyuan, Taiwan; Graduate Institute of Basic Medical Science, China Medical University, Taichung, Taiwan; College of Sports Science and Technology, Mahidol University, Salaya, Thailand; Department of Rehabilitation Science, China Medical University, Taichung, Taiwan; Department of Health and Nutrition Biotechnology, Asia University, Taichung, Taiwan; Department of Healthcare Administration, Asia University, Taichung, Taiwan

## Abstract

**Background:**

Ginsenoside Rb1, a principle active ingredients of *Panax ginseng*, has been shown to lower blood glucose in animals and increase insulin secretion in cultured insulinoma cells. The aim of this study was to determine the effects of daily ginsenoside Rb1 supplementation on circulating glucose and insulin levels in men during a 5-day recovery period after an acute bout of resistance exercise.

**Methods:**

Twelve gymnasts (20.5 ± 0.3 years of age) participated in this double blind placebo-controlled crossover trial. They were challenged by a lower-limb resistance exercise at a weight load of 85 % one-repetition maximal (1-RM) for 10 repetitions, six sets of the movement. Rb1 (1 ng/kg) or Placebo was orally delivered to participants daily during a 5-day recovery period after challenge. Circulating insulin, glucose and heart rate variability (HRV) were measured under fasted condition in the morning at Days 1, Day 3, and Day 5 during recovery.

**Results:**

No significant effect of Rb1 on circulating glucose and insulin levels were found among participants during the 5-day recovery period. A persistent elevation in sympathetic nervous activity, indicated by increased HRV-low frequency/high frequency (HRV-LF/HF) power, during the Rb1 trial was observed.

**Conclusions:**

The result of the study suggests that the null effect of Rb1 supplementation on lowering glucose and insulin levels of participants may be associated with chronic sympathetic activation.

## Background

Existing scientific literatures regarding glucose-lowering effect of ginseng in humans present conflicting results [1, 2]. Variation in ginsenoside profile associated with different species and cultivating season may account for the discrepancy among previous studies [1, 3]. Ginsenoside Rb1 is one of the most abundant ginsenosides in ginseng [4], which has been reported to stimulate insulin secretion in cultured Min6 cells [5] and decrease fasting glucose in rats [6]. To the best of our knowledge, no published data is currently available regarding whether oral Rb1 supplementation can affect circulating glucose and insulin levels in humans. Pancreatic insulin secretion and insulin sensitivity are, to some extent, modulated by autonomic nervous activity [7–10]. Despite effect of Rb1 on autonomic nervous activity is currently unknown, ginseng extract has been shown to influence the human autonomic nervous system reflected by heart rate variability analysis [11]. It has been reported that exercise containing eccentric contraction elicited a decreased insulin sensitivity in the whole-body glucose disposal [12]. The present work was undertaken to determine the effect of oral Rb1 supplementation on the glucose and insulin levels in athletes during a 5-day recovery period after a resistance exercise bout containing dynamic (concentric and eccentric) contraction of the lower extremities. Heart rate variability was recorded during a 5-day recovery period.

## Methods

### Subjects

Twelve male college gymnasts (aged 20.5 ± 0.3 y; height 169.4 ± 1.6 cm; weight 63.4 ± 1.8 kg) volunteered to participate in this study. Prior to the recruitment of volunteers, the study protocol was approved by Institutional Review Board of University of Taipei, Taipei, Taiwan. A detailed explanation of the study procedures, including the supplements to be received and potential risks might be involved, was informed to all participants prior to study. All subjects completed a written informed screening questionnaire after explanation. Participants had any form of health problem were precluded. All participants were asked not to change their dietary habit and not to participate in any form of training activity since a week before trial until the end of blood sample collection.

### Experimental design

This study used a placebo-controlled, double-blind, crossover design with a two-week washout period. Participants were randomly assigned to one of two parallel groups, initially in 1:1 ratio, to receive either Rb1 or Placebo under supervision of a lab staff to ensure subject compliance. The Rb1 and Placebo were made by the lab manager in liquid form and were identical in appearance. They were placed in a container and consecutively numbered for each participant according to the randomization schedule. Each participant was assigned an order number and received the drink in the corresponding container. A computer-generated list of random numbers was used for allocation of the participants. A research staff assigned participants to interventions. Except for the interventions, staff was kept blind to supplement assignment of the participants. Staff that takes outcome measurements and staff that delivers the intervention was different. Staffs and participants were maintained masked to outcome measurements and trial results. Before the experiment began, all participants engaged in parallel squatting and their 85 % maximum lower-limb muscle strength (85 % of 1-RM) was assessed. Baseline assessments was conducted under overnight fasted condition. Following a 30-min warm up period consisting of a 5-min warm up, a 10-min full-body stretching, a 5-min lower body stretching, a 5-min low intensity squat practice and a 5-min rest, all participants were challenged with a single bout of high-intensity lower-limb resistance exercise. In brief, this resistance exercise consisted of 6 sets of parallel squats with a resting interval of 60 s between sets [13]. Each exercise set comprised of a 10-parallel squat repetitions at 85 % one-repetition maximal (1-RM). All participants were instructed to complete each set of squat repetitions within 60 s.

### Rb1 supplementation

**Fig. 1:**
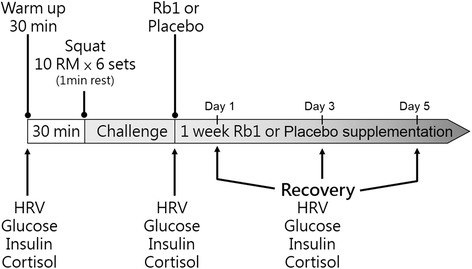
Experimental procedure. Post-exercise blood sample collection conducted before Rb1 or Placebo supplementation. Pre: before exercise; Post: after exercise; Day 1, Day 3, and Day 5: post-exercise recovery time with Rb1 or Placebo supplementation

Following exercise challenge, each participant was instructed to consume a solution containing ginsenoside Rb1 (or a placebo solution) for 5 consecutive days. Rb1 and Placebo drinks were made available by 0900 am in the morning. Participants orally received the drink once daily in the lab, under supervision of a staff at 0900 am during the 5-day recovery period. The Rb1, provided by NuLiv Science (Walnut, CA, USA), was extracted from Asian ginseng. Rb1 content is confirmed by Prof Tsu-Chung Chang in Department of Biochemistry, National Defense Medical College, Taiwan [14]. Rb1 crystal was solubilized by a 58 % alcohol solution into a concentrate. They were then withdrawn by pipetting into 50 ml of drinking water to achieve a final dose of 1 ng/kg body weight for each participants. The placebo group consumed same volume of drinking water, which contained an identical amount of alcohol solution. Participants were unable to tell the difference between Rb1 and Placebo drinks by appearance and taste. The experimental procedure is shown in Fig. 1. The dosage used in this study is equivalent to approximately 0.1 g of ginseng (wet weight), which is considered low dose.

### Samples collection

Blood samples were collected from fingertip and measured immediately for glucose concentration. Serum sample was used for insulin and cortisol measurements. A total of 200 μl of blood was collected from the fingertip for serum preparation, before and 10-min, 1 day, 3 day, and 5 days after resistance exercise challenge [13]. Each blood sample was centrifuged at 4000 rpm for 5 min. Supernatants were used as serum samples for insulin and cortisol measurements. They were frozen at −80 °C and analyzed within a week.

### Biochemical analysis

Fasting blood glucose was analyzed using a glucose-oxidase method with the One-Touch glucometer (LifeScan, Milpitas, CA, USA). All hormones were measured using commercially available enzyme-linked immunosorbent assay (ELISA) kits (Diagnostics Systems Laboratories, Inc., Webster, TX, USA) according to the standard procedures provided by the manufacturer. In brief, serum samples (depending on the requirements for specific kits) were loaded into 96-well plates coated with specific antibodies. Once antigens were bound with the specific primary antibody, the secondary antibody was added to form the antibody-antigen-antibody complex. The enzyme was then added and conjugated to the secondary antibody, and the plates were finally developed by adding enzyme substrate to generate visual light signals. The visual light signals were detected by ELISA analyzer (Tecan Genios, Salzburg, Austria). The intra-assay coefficient of variances (CV) for insulin and cortisol were 2.03 and 5.90 %, respectively.

### Heart rate variability (HRV)

HRV of all participants was assessed before (09:00), after exercise (11:50) and on the Day 1, Day 3 and Day 5 during recovery period (09:00). HRV was assessed in a quiet, dim environment at room temperature. Prior to assessment, participants were seated and instructed to relax for 5 min. An autonomic nervous system analyzer (Telemedicine Equipment Co., Ltd., Taiwan) was used to acquire a 5-min R-R interval data. HRV-HF was considered to be a measure of parasympathetic nervous system activity, whereas HRV-LF/HF was considered to be a measure of sympathetic nervous system activity [15].

### Statistical analyses

Two-way ANOVA with repeated measure (Rb1 supplementation and time) was performed to assess mean differences in all variables. Fisher’s least significant difference test was performed for post hoc comparison. Paired *t*-test was used to compare difference in area under the curve (AUC) of HRV-LF/HF. All values were expressed as mean ± standard error (SE). The statistical significance was set at 5 % of type I error.

## Results and discussion

**Fig. 2:**
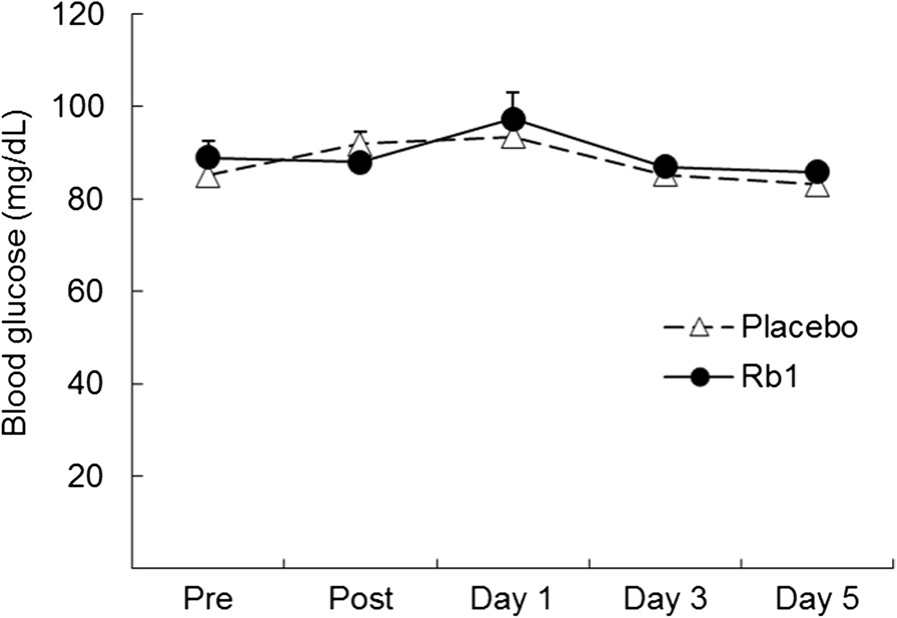
Blood glucose levels during a 5-day Rb1 supplementation after an acute bout of resistance exercise. All values are presented as mean ± SE. SI unit conversion factor from mg/dL to mmol/L: 0.0555

**Fig. 3:**
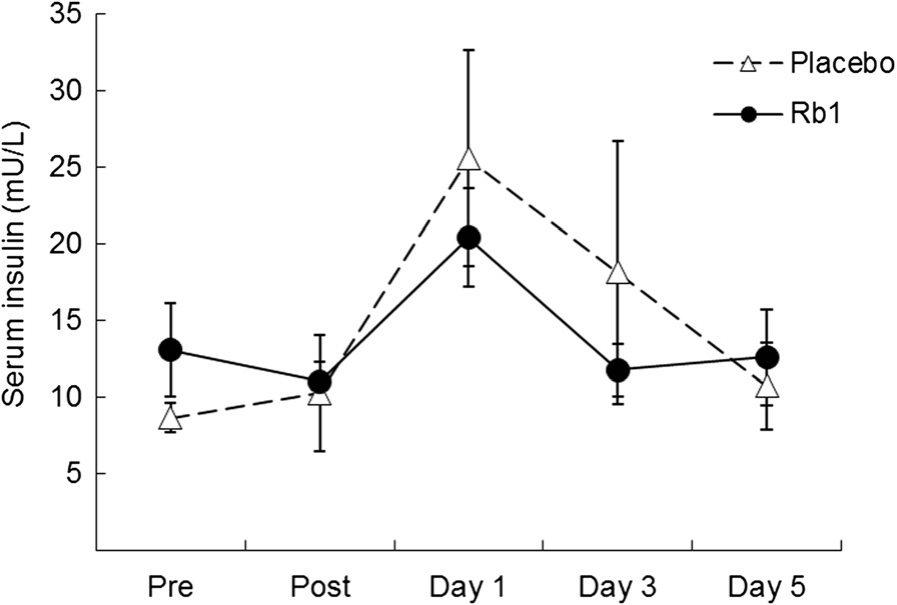
Serum insulin levels during a 5-day Rb1 supplementation after an acute bout of resistance exercise. All values are presented as mean ± SE. SI unit conversion factor from μIU/mL to pmol/L: 6.945

**Fig. 4:**
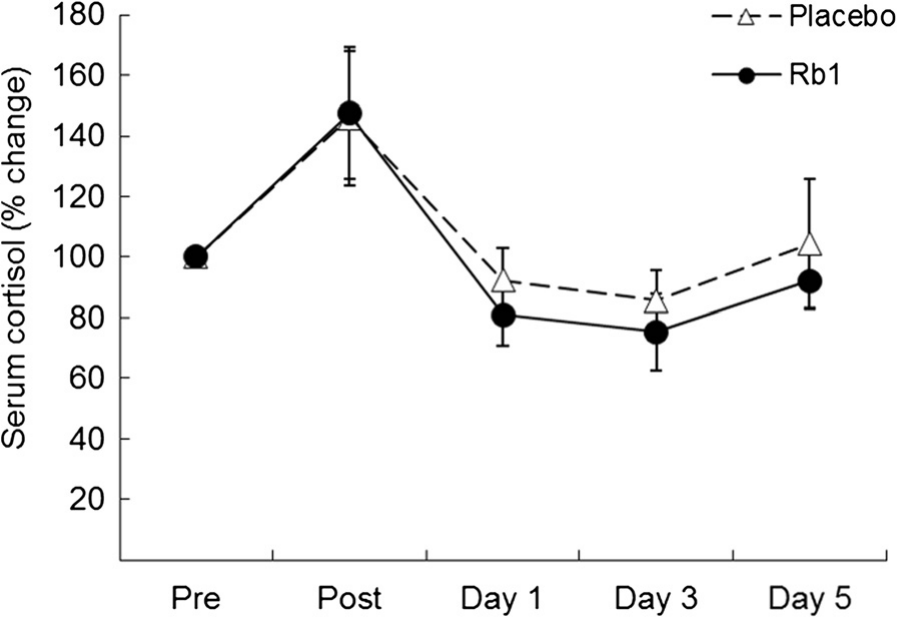
Serum cortisol levels during a 5-day Rb1 supplementation after an acute bout of resistance exercise. All values are presented as mean ± SE. SI unit conversion factor from μg/dL to nmol/L: 27.59

**Fig. 5:**
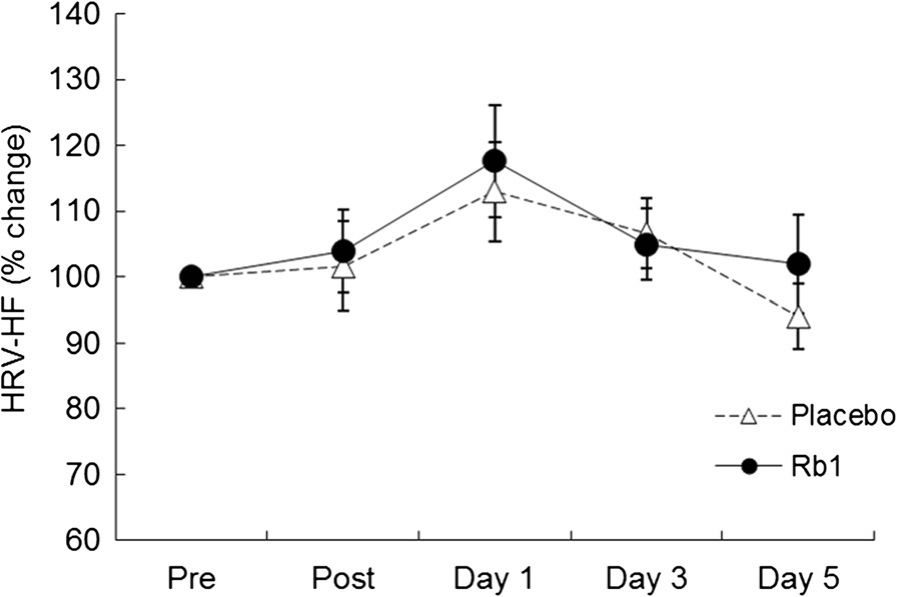
Vagal modulation (HRV-HF) during a 5-day Rb1 supplementation after an acute bout of resistance exercise. All values are presented as mean ± SE

**Fig. 6:**
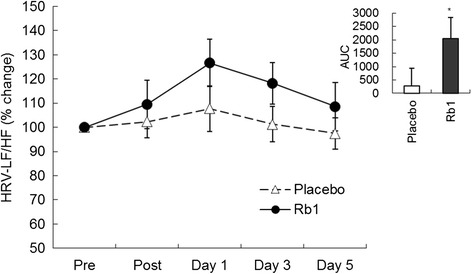
Sympathetic modulation (HRV-LF/HF) during a 5-day Rb1 supplementation after an acute bout of resistance exercise. AUC: Rb1 2036 ± 803 vs. Placebo 276 ± 655. *Significant trial effect (*P* < 0.05). All values are presented as mean ± SE

Timeline of experimental procedure and blood sampling is illustrated in Fig. 1. Time course data on circulating glucose and insulin are illustrated in Figs. 2 and 3, respectively. Both glucose and insulin concentrations between Rb1 and Placebo trials were not different throughout the 5-day recovery period. Cortisol data are illustrated in Fig. 4. No significant difference between trials was found during the 5-day recovery period. Effect of Rb1 on autonomic modulation is based on time course data from HRV analysis. Rb1 had no significant effect on vagal power (HRV-HF) during the recovery period (Fig. 5). However, sympathetic power (HRV-LF/HF) during Rb1 trial was significantly increased above Placebo level (Fig. 6). A significant trial effect was detected (*P* < 0.05).

We hypothesized that Rb1 would increase insulin and decrease glucose in men after a resistance exercise, based on the existing data showing lowered blood glucose in rats [16] and increased insulin secretion in cultured insulinoma cells [5] after Rb1 treatment. However, we did not detect significant change in circulating glucose and insulin during the 5-day Rb1 human trial. Our result is similar to two studies using crude material of ginseng, which shows no improvement in glucose metabolism of cyclists for a 7-day Rb1 trial [17, 18]. Given the fact that Rb1 is a major component of ginseng, the result of the study implicates that Rb1 from ginseng cannot influence glucose and insulin in humans during an exercise recovery at this dose.

Sympathetic activation by Rb1 may be one reason to explain such unanticipated outcome for the absence of glucose-lowering effect. Increased sympathetic activity is generally associated with reduced insulin sensitivity in humans [8], which is also supported by a causal relationship of an increased insulin sensitivity by sympathetic inhibition in obese patients [19]. Ginseng is known as a modulator for the autonomic nervous system [11]. Thus, result of the present study on persistent sympathetic activation suggests that Rb1 is an active component of ginseng and mediated its action via autonomic modulation, which masks our observation on glucose lowering action of Rb1.

Despite ginseng action on glucose metabolism being widely studied subject for human use, most available data come from studies using raw material or extract. One major barrier to establish the knowledge regarding whether ginseng can be designed as a hypoglycemic agent is the variation of ginseng source. In a double-blind, randomized, multiple-crossover trial, healthy participants received the same amount of American, American-wild, Asian, Asian-red, Japanese-rhizome, Vietnamese-wild, Sanchi, Siberian ginsengs and Placebo, and showed mixed outcomes on glucose tolerance [1]. Ginseng from the same cultivated land but different batch can also generate inconsistent outcomes on glucose tolerance in humans [3]. Such discrepancy is presumably associated with the changing ginsenoside profile due to season, suggested by the observation that ineffective ginseng batch on improving glucose tolerance also contains decreased ginsenoside content including Rb1 than those effective ginseng batch.

Despite the result of our study does not favor Rb1 as a hypoglycemic component of ginseng as suggested by aforementioned animal studies, we do not preclude the possibility that this ginsenoside component of ginseng may have its claimed action at different doses. The current dose of the study for our human participants was 1 ng/kg. Most of the effective doses tested in previous animal studies are in the range between 5–60 mg/kg, which is substantially greater than what we have been used in the present study [6, 20, 21]. Due to this limitation, dose–response human trial would be needed for further clarification.

Ginseng supplementation has been reported to decrease cortisol levels in humans [22–24]. Since cortisol is a potent stress hormone that stimulates hepatic glucose output and causes peripheral insulin resistance, we were expecting a decrease of cortisol by Rb1 treatment. However, no significant change was detected during the 5-day human trial.

## Conclusions

The present study asks the question whether ginsenoside Rb1 can influence systemic glucose or insulin levels in humans after an acute bout of resistance exercise, based on the findings of increased insulin release in pancreatic cells and decreased blood glucose level in rats. However, our data did not find any changes in circulating glucose and insulin levels with a low dose Rb1 supplementation. This result may be explained by a persistent elevation in sympathetic nervous activity by Rb1 supplementation.
